# A Retrospective Study of CBCT-Based Detection of Endodontic Failures and Periapical Lesions in a Romanian Cohort

**DOI:** 10.3390/jcm14186364

**Published:** 2025-09-09

**Authors:** Oana Andreea Diaconu, Lelia Mihaela Gheorghiță, Anca Gabriela Gheorghe, Mihaela Jana Țuculină, Maria Cristina Munteanu, Cătălina Alexandra Iacov, Virginia Maria Rădulescu, Mihaela Ionescu, Adina Andreea Mirea, Carina Alexandra Bănică

**Affiliations:** 1 Department of Endodontics, Faculty of Dentistry, University of Medicine and Pharmacy of Craiova, 200349 Craiova, Romania; oanamihailescu76@yahoo.com (O.A.D.); leliagheorghita@yahoo.com (L.M.G.); ancaggheorghe@gmail.com (A.G.G.); mtuculina@yahoo.com (M.J.Ț.); karinabanica@yahoo.com (C.A.B.); 2 Department of Oral and Maxillofacial Surgery, Faculty of Dental Medicine, University of Medicine and Pharmacy of Craiova, 200349 Craiova, Romania; cristina_omf@yahoo.com; 3 Department of Medical Informatics and Biostatistics, Faculty of Medicine, University of Medicine and Pharmacy of Craiova, 200349 Craiova, Romania; virginia.radulescu@umfcv.ro (V.M.R.); mihaela.ionescu@umfcv.ro (M.I.); 4 Department of Oral Prevention, Faculty of Dental Medicine, University of Medicine and Pharmacy of Craiova, 200349 Craiova, Romania

**Keywords:** cone-beam computed tomography (CBCT), apical periodontitis (AP), retrospective studies, root canal filling materials, root apex

## Abstract

**Background and Objectives**: Cone Beam Computed Tomography (CBCT) offers high-resolution, three-dimensional imaging for detecting apical periodontitis (AP) and evaluating the technical quality of endodontic treatments. This study aimed to investigate the diagnostic value of CBCT in identifying endodontic failures and periapical lesions and to explore the clinical patterns associated with these findings in a Romanian patient cohort. **Materials and Methods**: A retrospective study was conducted on 258 patients (with 876 root canal-treated teeth), all of whom underwent CBCT imaging between October 2024 and April 2025 at a private radiology center in Craiova, Romania. Of the 876 treated teeth, 409 were diagnosed with apical periodontitis. Patients were present for endodontic treatment at the Endodontics Clinic of the Faculty of Dentistry, University of Medicine and Pharmacy of Craiova. With the patients’ consent, 3D radiological examinations were recommended for better case planning and accurate diagnosis. The periapical status and technical parameters of root canal fillings were assessed using the CBCT-PAI index and evaluated by three calibrated observers. Associations with demographic, clinical, and behavioral factors were statistically analyzed. **Results**: Apical periodontitis was detected in 46.69% of the teeth examined during the study period, with CBCT-PAI score 3 being the most prevalent. Poor root canal obturation quality (underfilling, overfilling, and voids) was significantly associated with periapical pathology. Chronic lesions were more common than acute ones, especially in older patients. The number of teeth with endodontic treatments and no AP, as well as the number of teeth with AP, was significantly lower for patients with acute AP, indicating the more severe impact of chronic AP on the patients’ oral health status. CBCT allowed the precise localization of missed canals and assessment of lesion severity. **Conclusions**: Within the limits of a retrospective, referral-based cohort, CBCT aided the detection of periapical pathology in root canal-treated teeth (46.69%). These findings do not represent population-based rates but support the selective use of CBCT, in line with current ESE guidance, for complex cases or when conventional imaging is inconclusive.

## 1. Introduction

Apical periodontitis is a common consequence of inadequate root canal filling and remains a major clinical challenge in endodontics. Despite technological advances and improved protocols, the prevalence of suboptimally treated teeth continues to rise [1,2]. Numerous studies have demonstrated a strong association between failed endodontic therapy and the presence of apical pathology [3], emphasizing the need for accurate diagnosis and quality control throughout the treatment process.

The persistence or recurrence of apical periodontitis is often linked to residual infection in the root canal system, procedural errors, or inadequate obturation [4,5,6,7]. Radiographic evidence of periapical radiolucency is typically associated with treatment failure, often due to missed canals, poor aseptic control, or suboptimal coronal restorations [8,9,10,11,12].

Endodontic treatment aims to eliminate infection and prevent reinfection, but persistent apical lesions remain a frequent complication, especially in technically deficient cases [13,14,15]. Conventional radiographic methods—although widely used—offer limited sensitivity in detecting early periapical changes or evaluating complex anatomy [16,17].

Cone Beam Computed Tomography (CBCT) has emerged as a valuable diagnostic tool, providing three-dimensional high-resolution images of root canal anatomy and periapical lesions [18,19,20]. CBCT demonstrates superior sensitivity compared to periapical radiographs in detecting untreated canals, fractures, voids, overfills, and subtle apical bone destruction [21,22,23,24,25]. This enhanced diagnostic capability has been validated in numerous studies and meta-analyses [26,27,28], especially in complex cases requiring retreatment or differential diagnosis. Furthermore, studies such as those by Abella et al. and Del Fabbro et al. support the clinical accuracy and reliability of CBCT in endodontic diagnostics and retreatment planning [29,30].

Despite these advantages, CBCT must be used selectively and judiciously. According to the 2023 clinical practice guideline of the European Society of Endodontology (ESE), CBCT is not recommended for routine follow-up but should be reserved for situations where conventional imaging fails to provide sufficient diagnostic information [5,20].

While previous studies have assessed the prevalence and severity of apical periodontitis using CBCT and CBCT-PAI [20,28], few have explored correlations with technical, clinical, and behavioral risk factors in Eastern European populations. Thus, this study aimed to evaluate the periapical status and root canal filling quality in a Romanian patient cohort using CBCT imaging and to identify associations with systemic and demographic variables.

We formulated the null hypothesis that the occurrence of apical periodontitis is not significantly associated with age, gender, systemic health conditions, harmful habits, or root canal filling quality.

Therefore, the primary objective of this study was to evaluate the association between apical periodontitis (AP) and the technical quality of root canal fillings as assessed by CBCT. The primary endpoint was the presence or absence of apical periodontitis, based on the CBCT-PAI score. We hypothesized that inadequate root canal obturation (underfilling, overfilling, or voids) would be significantly associated with the presence of AP.

## 2. Materials and Methods

### 2.1. Study Design

This retrospective, cross-sectional observational study was conducted between October 2024 and April 2025 on a cohort of patients from the southwestern region of Romania. Data collection was based on CBCT scans and corresponding clinical records from patients evaluated during this period. All data were already available in the radiology center’s database at the time of analysis, and no prospective follow-up was required.

The timeframe (October 2024–April 2025) refers exclusively to the dates of CBCT acquisition and clinical diagnosis; no longitudinal follow-up or outcome monitoring was performed. Data analysis began only after the inclusion period was completed, once all imaging and diagnostic information had been centralized.

Therefore, there was no delay between the end of the inclusion period and data analysis. The patients included in the study required endodontic treatments, which were performed at the Endodontics Clinic of the Faculty of Dentistry, UMF Craiova. Following patient evaluation and clinical diagnosis, data were centralized retrospectively from the clinical files and CBCT database for analysis. For a more accurate diagnosis and better case planning, preoperative CBCT scans were performed at a private radiology center in Craiova. In some asymptomatic patients, CBCT was also prescribed but only when conventional 2D imaging (periapical or panoramic radiographs) was inconclusive or insufficient for safe and effective treatment planning. Typical indications included suspected missed canals, complex root canal morphology, or ambiguous findings following prior endodontic treatment. This selective prescription of CBCT is consistent with the ESE 2023 guidelines, which recommend its use exclusively when additional diagnostic information is essential for clinical decision-making. Furthermore, it adheres to ethical principles of radiation protection, including the justification requirement and the ALARA principle, ensuring that patient exposure was warranted in all cases [5].

The study was approved by the Ethics Committee of the University of Medicine and Pharmacy of Craiova (Approval No. 108/27.01.2025), and all procedures adhered to ethical and legal standards. This retrospective study involved no direct patient contact or clinical intervention. All CBCT scans and associated clinical records were anonymized before analysis. Written informed consent for the use of anonymized data in research was obtained from all patients as part of the standard clinical and imaging documentation at the time of care, in accordance with institutional protocols and ethical regulations.

Although the CBCT scans used in this study were acquired between October 2024 and April 2025, no data analysis or access to patient records occurred before ethical approval was obtained on 27 January 2025. The Ethics Committee had been notified of the study intention prior to the imaging period, and the delay in formal approval was due to high submission volumes from institutional researchers.

The study included patients referred for CBCT evaluation of root canal-treated teeth, regardless of the presence or absence of symptoms. All participants had at least one previously endodontically treated tooth, clinical documentation, and CBCT images of diagnostic quality. The CBCT scans included in this study were obtained selectively—primarily for advanced treatment planning or in cases with suspected complications. While this approach aligns with ESE guidelines, it introduces a selection bias, as the cohort is enriched with complex or failure-prone cases. As a result, the observed prevalence of apical periodontitis may overestimate rates found in the general population of root canal-treated teeth. These findings should be interpreted in the context of a high-risk, referred patient population. This limitation affects the external validity of the study and is acknowledged accordingly [5].

The minimum required sample size regarding the number of patients was calculated using G*Power 3.1.9.7 (Heinrich Heine University Düsseldorf, Düsseldorf, Germany), for a χ^2^ test family, based on a significance level of α = 0.05, statistical power of (1 − β) = 0.95, and an effect size of 0.25, resulting in a minimum of 248 participants. Since this is a retrospective study, this calculation ensures that the findings of the present research concerning the patients are reliable and generalizable.

The inclusion criteria were as follows:

- Patient age ≥ 17 years;
- CBCT imaging performed during the study period;
- Presence of at least one previously endodontically treated tooth;
- Complete clinical documentation;
- High-resolution CBCT images suitable for diagnostic analysis.

The exclusion criteria included the following:

- No history of root canal therapy;
- Teeth with ongoing or incomplete endodontic treatment at the time of imaging;
- CBCT images with significant artifacts, poor resolution, or anatomical distortion;
- Third molars, due to anatomical variability and limited diagnostic relevance;
- Severely damaged coronal structures preventing proper assessment of root fillings;
- Patients who did not consent to the research use of anonymized data.

In asymptomatic cases, CBCT examinations were performed only when conventional periapical radiographs were inconclusive or insufficient for treatment planning, such as in cases with suspected missed canals, complex root morphology, or previously failed endodontic treatment. This selective approach is consistent with the ESE 2023 guidelines, which recommend CBCT use only when additional diagnostic information is essential for clinical decision-making [5].

From the records, the following parameters were collected:

- Demographic: age, gender;
- Systemic health: presence of chronic diseases;
- Behavioral: harmful habits (e.g., smoking);
- Dental variables: total number of teeth, number of endodontically treated teeth, presence of abutment teeth or intraradicular posts, dental arch affected, and tooth type;
- Technical parameters: obturation length (underfilling, overfilling, correct), quality (gaps, voids), presence of missed canals.

The quality of endodontic treatment was assessed based on the three-dimensional CBCT appearance of the root filling and periapical status. All CBCT scans and clinical records were retrospectively reviewed; data were fully available at the time of analysis and no prospective follow-up was required.

### 2.2. Observer Calibration and Reliability Assessment

Three calibrated endodontists, each with over 10 years of clinical experience, independently evaluated the CBCT scans. Calibration was performed on a separate set of 20 randomly selected images. Inter- and intra-observer reliability was assessed using the intraclass correlation coefficient (ICC) calculated via a two-way mixed-effects model for absolute agreement. ICC values ranged from 0.939 to 0.989, indicating excellent agreement; however, the small number of images used for calibration may limit the robustness of these estimates.

### 2.3. CBCT Imaging Parameters

CBCT images were obtained using a Carestream CS 8200 3D (Carestream Dental LLC, Atlanta, GA, USA) with a voxel size of 75 μm and a 5 × 5 cm field of view. Exposure parameters were 90 kV, 10 mA, and 10.8 s, following the manufacturer’s endodontic imaging protocol.

The images were assessed using CS 3D Imaging version 8 Software (Carestream Dental), which allows for multi-planar reconstruction, axial, coronal, and sagittal views, as well as volumetric rendering. All evaluations were performed under standardized lighting conditions on calibrated high-resolution monitors.

**Figure 1:**
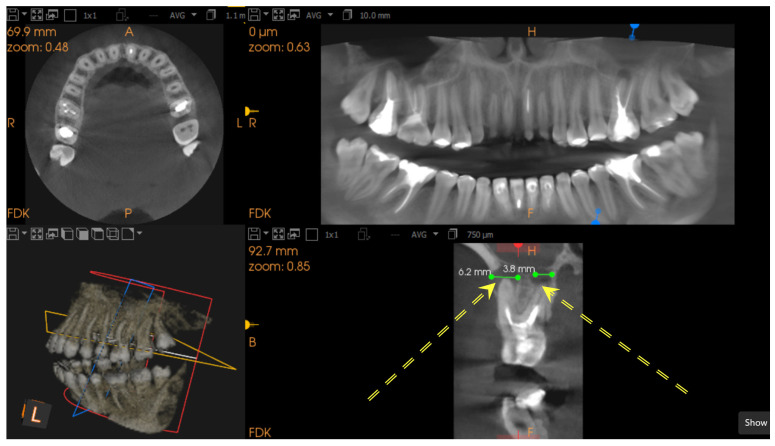
Multiplanar CBCT images of a maxillary molar with periapical radiolucency measuring 3.8 mm and 6.2 mm. The lesion (marked by double yellow arrows) is well-circumscribed and consistent with a CBCT-PAI score of 3–4. Views include axial, panoramic reconstruction, 3D rendering, and sagittal slice.

**Figure 2:**
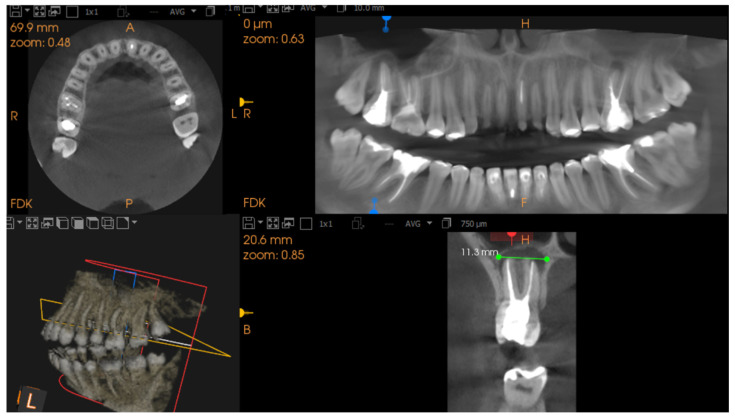
CBCT images demonstrating an extensive periapical lesion of 11.3 mm (marked by double yellow arrows) with poorly defined margins, consistent with a CBCT-PAI score of 5. The multiplanar view allows assessment of lesion dimensions and involvement of surrounding structures.

Diagnosis of apical lesions was based on the CBCT Periapical Index (CBCT-PAI) [19]. A healthy periapical region was defined by the absence of radiolucency. The presence of radiolucency indicated apical periodontitis. CBCT-PAI was determined by the extension of the lesion. Only CBCT-PAI scores 3, 4, and 5 were identified and included in the analysis [20]. Representative examples are shown in Figure 1 and Figure 2.

The CBCT-PAI scoring system (scores 3–5, in our study sample) was used to assess the severity of periapical radiolucencies, given that no scores of 1 or 2 were identified.

Root canal treatment quality was evaluated based on criteria from the European Society of Endodontology, including apical extent and density of canal fillings. Root filling quality was classified as satisfactory (homogeneous, well-adapted) or unsatisfactory (voids, over- or under-filling) [20].

Apical limit of the canal filling:

- Correct root canal filling, the apical limit of the root canal filling at 0–0.5 mm from the radiological apex;
- Incorrect root canal filling, the root canal filling 2 mm smaller than the radiological apex (short fillings, underfillings) and the root canal filling exceeding the radiological apex by 2 mm (overfillings).

Density and homogeneity of the root canal filling:

- Satisfactory root canal filling—dense, homogeneous density, adapted to canal walls;
- Unsatisfactory root canal filling: uneven filling, visible gaps between filling and canal wall.

The diagnosis of chronic versus acute apical periodontitis (CAP vs. AAP) was based primarily on the preoperative clinical records documented by the treating clinicians. For each case, we reviewed the patient’s medical file to extract the clinical diagnosis, and we used the CBCT images to assess radiological consistency with that diagnosis.

Chronic apical periodontitis was characterized radiographically by a well-circumscribed radiolucency with corticated borders, suggestive of a long-standing, asymptomatic inflammatory process. In contrast, acute apical periodontitis presented as an ill-defined radiolucency, often lacking cortication and sometimes showing bone rarefaction extending into the surrounding trabecular bone.

While imaging descriptors were applied, they served to illustrate lesion morphology, not to independently define the acute/chronic status.

**Figure 3:**
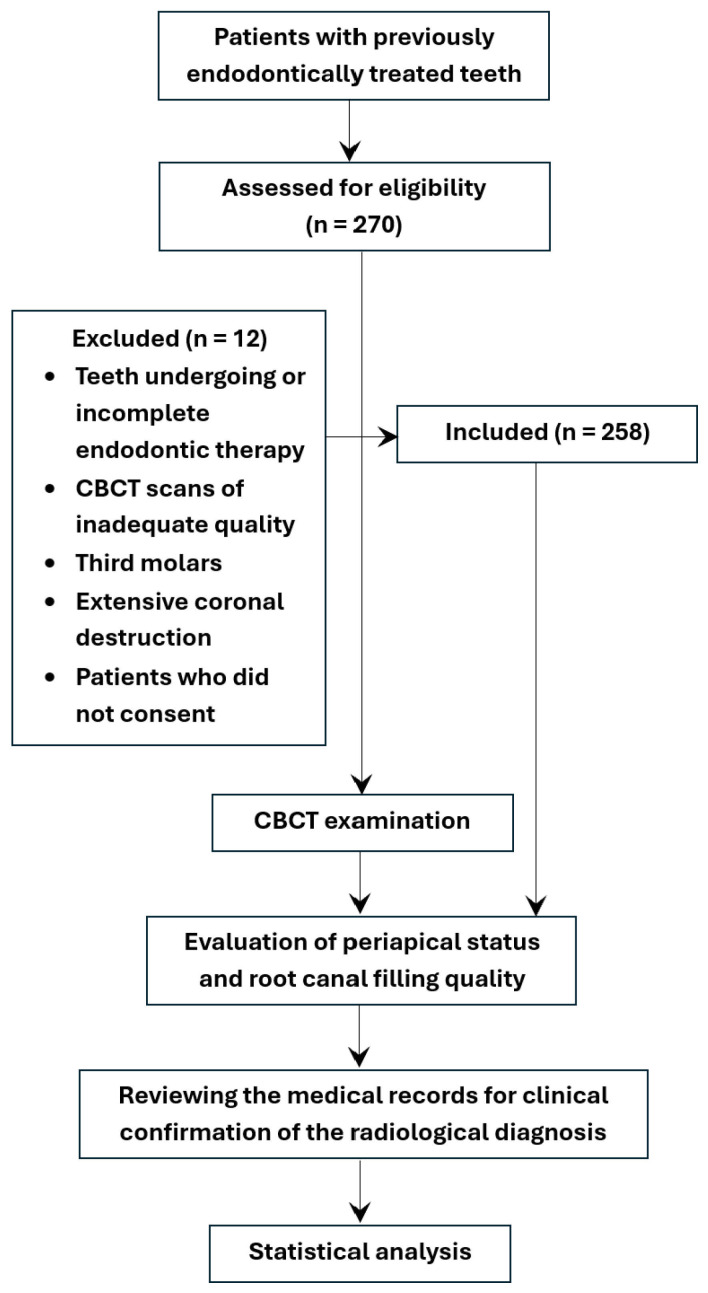
Flowchart illustrates the study methodology, including patient selection, inclusion/exclusion criteria, and CBCT evaluation process.

All data obtained from the study were centralized, electronically filed and statistically analyzed, as in Figure 3.

### 2.4. Statistical Analysis

Primary data were first organized and categorized for statistical analysis using Microsoft Excel 2021 (San Francisco, CA, USA). The data were then categorized into subgroups based on the type of analysis required. For the statistical analysis, we used IBM’s Statistical Package for the Social Sciences (SPSS), version 26.0 (IBM Corp., New York, NY, USA). Normality of continuous variables was assessed using the Shapiro–Wilk test, supplemented by scatter plots. Continuous variables were expressed as the mean ± standard deviation (SD) for suggestive comparisons and an easier understanding, as well as median values within statistical comparisons for series without a normal distribution. Nominal data and ordinal variables were presented as absolute and relative frequencies (%). For data that did not follow a normal distribution, associations were examined using the Mann–Whitney U test, and the effect size, denoted as *r*, was computed as the ratio between the absolute value of the standardized test statistic z and the square root of the number of observations in the dataset (the sample size). Associations between nominal variables were tested using the Chi-square test; the magnitude of the association between these variables, or the effect size, was determined as the ratio between the square root of the chi-square value and the sample size. Statistical significance was established based on an alpha threshold set at 5%, with *p*-values less than 0.05 indicative of significance, and a confidence interval maintained at 95%.

## 3. Results

### 3.1. Patients’ Analysis

The initial analysis aimed to characterize the demographic and clinical structure of the study group, with a focus on gender, age distribution, general health status, and lifestyle risk factors. Gender distribution showed a slight female predominance, with 145 women (56.20%) and 113 men (43.80%) included in the cohort. Patients’ ages varied from 17 to 84 years old, mean age 42.88 ± 15.39 years old, and exhibited a non-Gaussian distribution. To help the clinical interpretation of demographic and behavioral characteristics, patients were grouped by gender and age using 40-years old as a cutoff. This threshold was selected due to its clinical relevance, as it roughly delineates the transition between early adulthood and midlife—a period frequently associated with the emergence of cardiometabolic risk factors and the onset of chronic systemic conditions. Also, the age of 40 is frequently mentioned in screening guidelines and risk assessment protocols, which reinforce this two-group classification. In addition, patients’ ages were not normally distributed, and the median value of the series indicating the series’ tendency was 40.

Gender analysis revealed no statistically significant differences between females and males regarding the demographical and general medical factors (Table 1). The median age was higher in women (44.00 years) compared to men (37.00 years), but this difference did not reach statistical significance (*p* = 0.129). When patients were further grouped into two age categories (≤40 and >40 years old), the proportions remained relatively balanced across genders, with no significant association identified (*p* = 0.285). These findings indicate a demographically homogeneous sample in terms of both continuous and categorical age distributions (Table 1).

Although the numbers and distributions of chronic medical conditions are higher for females, statistical analyses failed to find any significant relationship between a participant’s gender and the incidence of chronic diseases (*p* = 0.116). Overall, the two groups, women and men, appear to be equally well off as they shared a similar burden of chronic disease, and there were no real differences underpinned by gender. A borderline value was computed for the difference between genders regarding the total number of teeth: median value 26.00 for females compared to 27.00 for males, *p* = 0.052, taking into account the fact that patients from the older age groups were predominantly females.

Overall, females have more teeth with endodontic treatments compared to males, median values 3.00 and 2.00, respectively; however, this difference did not reach the statistical significance threshold. Similarly, females had more endodontically treated teeth with no AP lesions, compared to males, but the differences were not statistically significant (*p* = 0.082). In terms of the number of teeth with AP lesions, both acute and chronic, both genders showed very similar values. No other statistically significant differences were identified between genders (Table 1).

Age analysis revealed that chronic diseases are mostly present in the elderly group, being present for 58.54% of patients over 40 years old, compared to just 3.70% for younger patients (≤40 years old), the differences being statistically significant, *p* < 0.0005. Abutment teeth and intraradicular posts are also present in a more significant number in elderly patients, compared to younger patients, *p* < 0.05, Table 2.

The overall status analysis of the young participants indicated that this subgroup is characterized by a generic better health status, with a statistically significantly higher total number of teeth, and a significantly lower number of teeth with endodontic treatment, the number of teeth endodontically treated and without AP lesions, number of teeth with AP lesions, both acute and chronic, *p* < 0.05 (Table 2).

The assessment of periapical diagnosis and canal filling characteristics indicated some clear patterns of significance based on the type of AP (Table 3).

The distribution of AP lesions was initially analyzed as stratified by gender and age group, to evaluate whether demographic criteria had an effect on the distribution of periapical pathosis. In the entire cohort in this study, most patients had just one apical lesion, with a similar pattern in both sexes and age groups. Particularly, 52.00% of females had one lesion and 50.40% of males had one lesion, which seems to indicate a general trend toward more localized periapical pathosis. Chronic AP was the most prevalent diagnosis found in both women and men (183 patients), with rather similar rates of occurrence, indicating that both sexes are afflicted by this condition. Conversely, acute AP did seem to occur slightly more in female patients (60.00%) than in males (40.00%); however, this difference was not significant (*p* = 0.431).

The proportion of patients with one AP lesion was slightly higher in the ≤40 years category for females (31.10%) and males (30.40%) than in older patients (20.90% and 20.00%, respectively). Specifically, while only a small increase in the frequency of patients with multiple AP lesions (≥2) was observed in the >40 years group (which was primarily for females), the proportion of patients with three AP lesions had increased to 7.10%, from 3.70% in patients aged ≤40 years old. Acute AP affected mostly younger patients (with 65.33% of all patients with acute AP), while the status for chronic AP was quite opposite, with a higher prevalence in elderly patients. For both age groups, analyzed individually, chronic AP was more frequent than acute AP, with percentages above 60%. Overall, the difference between age groups related to the type of AP lesions was statistically significant, *p* = 0.007 (Table 3). As the presence of medical conditions is related to the age group, a statistically significant difference between patients with various health status and AP type was also identified: 80.00% of patients with chronic diseases also have chronic AP, compared to only 66.85% of healthy subjects, *p* = 0.027.

Patients with acute AP had more teeth in the oral cavity, but fewer teeth with endodontic treatments, compared to patients with chronic AP, and this difference did reach the statistical significance threshold. Similarly, the number of teeth with endodontic treatments and no AP, as well as the number of teeth with AP was significantly lower for patients with acute AP, indicating the more severe impact of chronic AP on the patients’ oral health status, a fact supported also by the predominant presence of abutment teeth and intraradicular posts for chronic patients (*p* < 0.05).

Chronic AP was present for more than 90% of patients with both arches affected by AP. This percentage decreased to 67.50% for patients with AP identified at the superior arch level, and decreased even more, to 43.10%, for patients with AP at inferior arch level, *p* < 0.0005.

Technical aspects of root canal filling demonstrated several significant associations. More than 70% of patients with chronic AP had underfillings and overfillings, while the percentage of patients with acute AP was significantly lower. Correct root canal obturations were also predominant for patients with chronic AP, but not statistically significantly higher than for patients with acute AP. Underfillings were significantly more frequent in chronic AP (73.82%) compared to acute AP (26.18%) (*p* = 0.002). Overfilled canals followed a similar trend, with a significantly higher prevalence in chronic cases (87.27%) than in acute ones (12.73%) (*p* = 0.003). These findings may suggest that suboptimal root canal fillings are more likely to be associated with chronic forms, or that chronic inflammation develops more gradually in the presence of technical deficiencies. The size of the lesions in case of acute or chronic AP was similar, as well as the presence of gaps within the root canal filling, so they were without significant differences (*p* > 0.05).

These results emphasize the multifactorial nature of periapical pathology and underscore the importance of combining anatomical localization with technical quality indicators in the clinical assessment of endodontic outcomes.

### 3.2. Teeth Analysis

The 258 patients included in the study lot had a total number of 6408 teeth, and 876 teeth (representing 13.76% of the total number of teeth) were endodontically treated. Almost half of them (409 teeth, 46.69%) were diagnosed with AP based on CBCT-PAI criteria, with this being similar to the prevalence rates of apical periodontitis reported in other CBCT studies. Based on these values reflecting the real sample size, similar parameters as those used to compute the minimum patients’ sample size, the GPower analysis yielded an achieved power of the study of 0.949 for the *t* test family and 0.99 for the χ^2^ test family, which are considered acceptable for this present research. Patients contributed with more than one tooth; however, the majority of them had an average of 2 or 3 contributor teeth; an initial analysis regarding these variables, based on an intraclass correlation coefficient, yielded a very small variability for our studied parameters (which was considered acceptable by similar studies, so no clustering adjustment was applied, which may be considered a limitation of this study).

The analysis of demographic data revealed that the presence of AP was associated with all these factors. Gender differences were statistically significant within this study, as less than half of the females included in the study developed AP lesions, as opposed to males for whom more than half developed AP lesions, *p* = 0.013. Younger patients had more teeth with AP than older patients, and similar percentages were observed for healthy patients vs. patients with chronic diseases, *p* < 0.0005, Table 4.

All incisors included in the study had developed AP lesions, while less than half of premolars and molars (43%) had developed AP lesions, and the difference between these categories of teeth were statistically significant, *p* < 0.0005.

Technical factors related to the root canal filling are associated with the development of AP lesions. Almost 70% of teeth with satisfactory obturations had AP lesions, and this percentage decreased to 56.26% in the case of underfillings and to 33.33% for overfillings, leading to statistically significant differences between the different types of fillings, *p* < 0.0005. A satisfactory root canal filling was also associated with a high prevalence of AP lesions, *p* < 0.0005. Almost all teeth with undetected canals had developed AP lesions, while less than half of the other teeth were in this situation, and the differences were statistically significant, *p* < 0.0005 (Table 4).

According to Table 5, demographic factors had no influence on the quality of the root canal filling, *p* > 0.05, but the presence of harmful habits seemed to be associated with it, as 80.08% of teeth belonging to patients with harmful habits had an unsatisfactory treatment quality, a statistically significantly higher percentage compared to only 68.13% of teeth belonging to patients with no harmful habits, *p* < 0.0005.

The majority of molars (79.89%) had an unsatisfactory root canal filling, followed closely by incisors, with a percentage of 71.43%, and premolars with 66.43%; thus, the differences between teeth’ types clearly reached the statistical significance threshold, *p* < 0.0005. Similar results were obtained for the association between the presence of undetected canals and satisfaction of the treatment, *p* = 0.031 (Table 5).

Of the 409 teeth with AP lesions, 70.90% had developed small lesions, with low bone destruction (209 teeth), 28.60% had medium lesions, with moderate bone destruction (117 teeth), and 0.48% had large lesions, with severe bone destruction (2 teeth). For the ease of further calculations, given the small number of large lesions, teeth were divided into 2 subgroups: small lesions (CBCT-PAI score 3), and medium-large lesions (CBCT-PAI scores 4 and 5), values reflected in Table 6. These findings confirm the predominance of moderate periapical lesions in the sample, with severe forms less frequent but clearly present, particularly in older patients and posterior teeth. The absence of lower scores supports the notion that CBCT scans were predominantly prescribed for suspected endodontic failures or complex cases.

The AP lesion sizes revealed a distribution suggesting that female patients tended to present with more severe apical lesions compared to males. For example, PAI score 3 was recorded in 169 female patients (58.28%) versus 121 male patients (41.72%). Within genders, the distribution of PAI scores was also similar, with no statistically significant difference between females and males for overall PAI scores (*p* = 0.323). Age group and medical conditions distributions were also not statistically significant, indicating that demographic factors have no direct influence on the lesion size.

More than three-quarters of patients with harmful habits had small lesions, compared to 62.13% without these habits, leading to statistically significant differences between these groups, *p* = 0.001. Similarly, patients with acute AP tended to have more moderate to severe bone destruction (37.66%), compared to patients with chronic AP who had less medium-large lesions (27.11%), a fact not fully significantly justified by a borderline *p* value of 0.066. Almost two-thirds of premolars and one-third of molars had medium-large lesions, while half of the incisors had small lesions (53.06%); thus, the differences were statistically significant, *p* = 0.004.

Clear associations were identified for treatment quality and root canal quality, as 45.38% of teeth with overfillings had medium-large lesions, and this percentage decreased to 34.38% for teeth with correct treatment and to 19.84% for teeth with underfillings, leading to statistically significant differences between these groups, *p* < 0.0005. Half of the teeth with unsatisfactory root canal filling were characterized by moderate and severe bone destruction, compared to only 24.85% of teeth with a satisfactory level of treatment, thus indicating statistically significant differences, *p* < 0.0005.

AP lesion sizes did not seem to be associated with the presence of abutment teeth or intraradicular posts, the affected hemiarch, the presence of undetected canals, or the presence of gaps in the root canal filling, *p* > 0.05 (Table 6).

**Figure 4:**
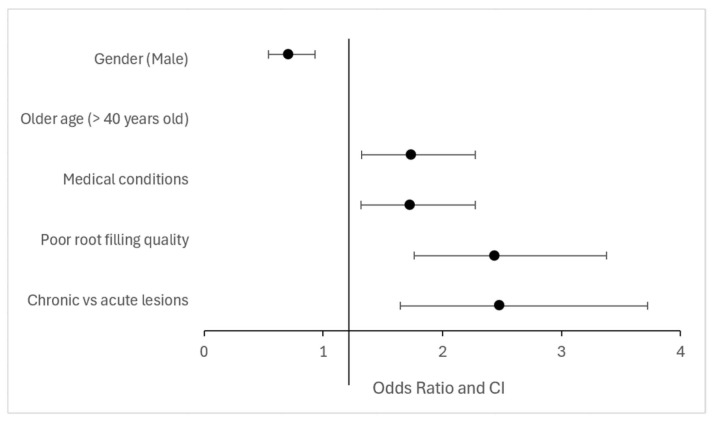
Key associations with apical periodontitis prevalence (odds ratio and confidence intervals).

A summary of the main associations between clinical/radiographic variables and the presence of apical periodontitis is provided in Table 7 and illustrated in Figure 4.

## 4. Discussion

The statistical results of this study support the rejection of the null hypothesis, demonstrating that the occurrence of apical periodontitis is significantly associated with patient age, gender, systemic medical conditions, harmful habits, and the technical quality of root canal obturations.

CBCT has demonstrated significant diagnostic advantages in detecting apical pathology compared to conventional radiography. Numerous studies support its superior sensitivity and specificity for identifying periapical bone defects, missed canals, root fractures, resorptive lesions, and technical complications such as under- and overfilled root canals [20,22,23,25,26,27,28,29].

In the present study, CBCT imaging revealed periapical lesions in 46.69% of teeth examined, a prevalence that is lower than the 63.5% reported by Estrela et al. [22] in a similar CBCT-based assessment. The discrepancy may be attributed to differences in population characteristics, sample size, or CBCT resolution.

Several associations approached the conventional significance threshold (e.g., *p* ≈ 0.05) and should be interpreted with caution, particularly given the tooth-within-patient dependence.

In our study, CBCT imaging revealed a high frequency of previously undiagnosed apical lesions and technical errors, including the presence of intraradicular posts, prosthetic abutments, and obturation deficiencies, all significantly associated with apical pathology. Moreover, CBCT-based data correlating endodontic pathology with systemic and behavioral conditions, such as diabetes, hypertension, and smoking. These findings confirm the value of CBCT not only as a diagnostic tool but also for optimizing clinical decision-making in complex cases. This trend mirrors observations made by Gomes et al. [29], who suggested a potential link between systemic health and endodontic outcomes, warranting further research.

Our cohort was demographically balanced across gender and age, with no statistically significant differences in the prevalence of chronic diseases or endodontic outcomes between men and women. These results align with previous reports that emphasize the limited role of gender as an independent risk factor in the development of periapical pathology [20,22]. Although women in our study had slightly more endodontically treated teeth than men, this trend did not reach statistical significance, consistent with findings by other authors [25].

Abella et al. argued that CBCT imaging reflects the true status of periapical tissues both before and after endodontic treatment [30]. In agreement with this perspective, the 2023 guidelines of the European Society of Endodontology recommend CBCT only for specific clinical scenarios where conventional radiography proves insufficient [5]. In line with ESE guidelines [5], we used CBCT selectively in diagnostically complex cases, consistent with these recommendations.

Traditional radiographic methods, although widely used, offer limited information in complex cases with overlapping structures or altered root anatomy, often leading to the underestimation of treatment errors [31].

While CBCT offers enhanced diagnostic capacity, its use must be justified to limit unnecessary radiation exposure [32].

Artifacts and noise in CBCT imaging can occasionally obscure fine details, especially in the presence of metallic restorations [33].

This study contributes novel CBCT-based epidemiological data for the Romanian population, which is currently underrepresented in endodontic literature. Although it does not introduce new diagnostic technologies, the study provides clinically relevant insights by correlating systemic and behavioral conditions—such as diabetes, hypertension, and smoking—with the presence of periapical pathology, as identified through high-resolution CBCT imaging.

Anatomical variations in the position of the apical foramen and the distance to the radiographic apex can influence both working length determination and the risk of over- or underfilling, which may explain part of the radiographically detected failures [34,35].

Treatment duration and number of appointments may also influence outcomes, though evidence remains inconclusive [35].

Orthograde retreatment remains a viable solution in cases of endodontic failure, provided that the cause—whether it is a missed canal or an underfilled root—can be accurately identified, often with the help of advanced imaging such as CBCT [36].

Although previous studies suggest that older adults may be less likely to undergo endodontic treatment due to cognitive or financial barriers [37,38], our findings showed frequent interventions among patients over 40. Specifically, treatments peaked in the 40–50 age group, coinciding with the period of active prosthetic rehabilitation [39,40], which may reflect increased clinical demand for tooth preservation driven by functional and aesthetic expectations.

Age, however, showed a more nuanced influence. When stratified by the clinically relevant 40-year threshold, older individuals exhibited more complex medical profiles and a higher number of treated teeth, although not always associated with AP lesions. These findings partially mirror the work of Duncan et al. [5], who noted that age-related changes in periapical tissues and systemic comorbidities may impact endodontic outcomes.

The relatively high prevalence of periapical lesions found in our study is consistent with CBCT-based investigations, where failure rates up to 73% have been reported [41,42]. Such variability in prevalence is often due to differences in imaging modalities and population characteristics, reinforcing the diagnostic value of CBCT for accurate outcome assessment.

While gender was not directly associated with the presence of periapical lesions in our cohort, it was significantly linked to technical parameters such as underfilling and overfilling—both of which are known contributors to apical pathology. These findings highlight the importance of considering indirect associations when analyzing endodontic outcomes.

Regarding technical quality, our study identified 33.5% of root canals as underfilled and 12.7% as overfilled. These findings closely parallel those of Jiménez-Pinzón et al. [23], who observed inadequate filling lengths in 37% of cases. This reinforces the persistent clinical challenge of achieving optimal obturation.

Our study confirmed that patients under 20 years of age had the lowest prevalence of periapical lesions, consistent with previous literature [32,42,43,44,45]. Conversely, a progressive increase in lesion frequency was observed with advancing age, particularly in individuals over 40—a trend also supported by the statistical analysis of our cohort.

In our study, molars—particularly mandibular molars—were the most frequent site of periapical pathology, followed by premolars. This distribution supports previous findings that highlight the increased risk of endodontic failure in posterior teeth due to their complex root canal anatomy, difficult access, and higher masticatory load [46]. However, this contrasts with other studies, such as those by Gulsum [39] and De Moore [45,46], which reported higher prevalence in anterior maxillary incisors. These discrepancies may be due to differences in population demographics, diagnostic imaging methods, and inclusion criteria.

CBCT imaging has proven particularly useful in identifying accessories or untreated root canals, which are often missed in conventional radiographs [47].

Missed canals were noted in 6.4% of our cases, a figure comparable to that reported by Abella et al. [30], who found untreated canals in approximately 7% of teeth using CBCT. These findings support the role of three-dimensional imaging in identifying complex root anatomy and improving treatment planning.

Vertical root fractures, which are notoriously difficult to detect on 2D imaging, can be accurately visualized using CBCT, which enhances clinical decision-making [48].

The presence of metallic posts, often associated with technical limitations and CBCT artifacts, was observed in 8.6% of teeth in our cohort. Kamburoglu et al. [33] reported similar diagnostic limitations, emphasizing the need for the cautious interpretation of scans in such cases.

Several studies have demonstrated the superior sensitivity of CBCT in detecting periapical lesions compared to periapical radiographs, especially in molars and multi-rooted teeth [49].

Despite its advantages, CBCT is not without limitations, such as beam hardening artifacts and higher radiation dose, which must be carefully weighed in clinical indications [50].

There is a strong correlation between inadequate root canal obturation and the presence of apical periodontitis, as emphasized in recent imaging-based studies [51].

Failure of endodontic treatment is often associated with complex root anatomy and canal systems that are difficult to detect without 3D imaging [52].

Studies have shown that apical lesions remain prevalent in root-filled teeth, underscoring the importance of both technical quality and anatomical factors [53].

This distribution likely reflects the greater anatomical complexity of molars and premolars, in contrast to anterior teeth, which typically present simpler canal configurations and higher success rates in primary endodontic treatment [54]. Extensive literature highlights the intricacy of posterior root canal systems, particularly in mandibular molars [55], mandibular premolars [56], and maxillary premolars [57], all of which are associated with an increased prevalence of missed canals and treatment failure.

Maxillary molars—particularly the mesiobuccal roots—are the most common sites of untreated canals, contributing significantly to endodontic failure [58]. Mandibular molars also present considerable clinical challenges in this regard, as shown by Kara-bucak [59]. CBCT enhances the preoperative identification of root canal systems, with studies reporting an average detection of 0.88 additional canals per previously treated tooth [36,60], underscoring its value in retreatment and surgical planning.

Our findings confirm that inadequate root canal obturation—whether underfilling or overfilling—is a primary contributor to treatment failure and the persistence of apical pathology. This is in line with extensive literature showing that residual intracanal infection often results from poor obturation quality [44,61,62,63]. Sunay et al. [64] reported that 90.8% of teeth with apical periodontitis had inadequate fillings, most commonly short. Similar trends have been observed with 74.3% of short fillings and 95.7% of overfilled cases presenting apical lesions [39].

Overfilled root canals are particularly problematic when associated with previous over-instrumentation, which may allow the extrusion of infected debris into the periapical tissues and compromise the apical seal [65].

The findings of this study align with the recent meta-analysis by a recent meta-analysis, hich identified poor obturation quality—particularly short or underfilled canals—as a key prognostic factor for treatment failure [66]. This association was consistently observed in our study, where inadequate root filling was significantly linked to apical periodontitis. However, regional differences may explain the slightly higher prevalence of apical lesions (46.69%) observed in our sample compared to the pooled data presented in the meta-analysis. These variations highlight the importance of context-specific assessments and the need for standardized evaluation criteria [66].

While our findings align with those of other studies regarding the negative impact of inadequate root canal fillings on periapical status, certain differences are noteworthy. The prevalence of apical periodontitis in our cohort (46.69%) is lower than in several studies included in the meta-analysis, which reported rates up to 68%. These discrepancies may reflect regional variations in treatment standards, differences in the imaging methods used (CBCT vs. conventional radiographs), and the selective nature of our sample. Unlike the meta-analysis, which incorporated heterogeneous data sources, our study relied exclusively on CBCT, allowing for higher diagnostic accuracy but also potential overestimation of lesion severity [66].

Obturations with inhomogeneous density have been consistently associated with microleakage and bacterial colonization, leading to persistent apical inflammation [36,67,68]. In our study, this relationship was statistically confirmed, with a highly significant association between obturation density and the presence of periapical lesions (*p* = 0.000), supporting existing literature.

Another key concern is the presence of internal voids within root fillings, which contribute to treatment failure. CBCT has been shown to be up to three times more sensitive than conventional radiography in detecting these defects [67]. Moreover, the size and irregularity of such intracanal cavities correlate directly with the severity of apical inflammation, as confirmed by several studies [69,70,71].

CBCT imaging also proved superior in detecting procedural complications related to inadequate primary endodontic treatments, including perforations, resorptions, separated instruments, and root fractures [26,72,73]. These diagnostic advantages are well documented in the literature and underscore the utility of CBCT in complex cases requiring retreatment or surgical planning [74,75].

Behavioral and psychosomatic factors may influence the development and persistence of apical periodontitis. In our study, smoking was not significantly associated with periapical lesions in male patients; however, harmful habits were more prevalent among older female patients. Prior research links smoking to impaired healing after endodontic retreatment [76,77], while anxiety, depression, and stress have been shown to affect both symptom perception and adherence to care [78].

All identified lesions exhibited moderate to severe apical bone loss on CBCT imaging. The absence of mild cases (scores 1 and 2) is consistent with the selective use of CBCT, which is typically reserved for patients with persistent symptoms or suspected endodontic failure [20,28].

The highest prevalence of CBCT-PAI score 3 was observed in both male and female patients, especially in the 21–40 and 41–60 age groups. A statistically significant association between age and CBCT-PAI score was found in female patients (*p* = 0.016), with lesion severity increasing progressively with age. This trend aligns with prior reports suggesting that a more severe pathology in older individuals may result from delayed diagnosis or subclinical inflammation [79,80].

In contrast, no significant age-related trend in CBCT-PAI scores was observed among male patients (*p* = 0.851), suggesting potential gender-based differences in disease progression or access to dental care. Similar CBCT-based studies have also noted stronger age–severity correlations in female patients [23,80,81].

The overall prevalence of apical periodontitis in our CBCT-evaluated sample was 46.69%, within the range reported in other CBCT-based studies [20,80], though higher than rates identified through conventional radiography.

Our findings support the use of the CBCT-PAI index not only as a diagnostic tool but also as a valuable aid in guiding therapeutic decisions, particularly in cases requiring endodontic retreatment. The results emphasize the importance of early diagnosis and meticulous treatment planning—especially in maxillary molars, where anatomical complexity often challenges clinical success.

Taken together, these findings reinforce the diagnostic and therapeutic value of CBCT, particularly in complex or uncertain clinical cases.

**Comparison with international data**

The prevalence of apical periodontitis (AP) in our referred CBCT cohort (46.69%) aligns with findings from larger CBCT-based analyses. A global meta-analysis—encompassing 639,357 teeth—reported a prevalence of AP of 39% (95% CI: 36–43%) in root-filled teeth and 5% (95% CI: 4–6%) overall [82]. Another CBCT study in Brazil found AP in 51.4% of patients and 3.4% of teeth [83]. A recent Saudi meta-analysis indicated AP prevalence of approximately 52% at the individual level and 5% at the tooth level; AP was 39% in root-filled teeth and 3% in untreated ones [84]. These findings confirm that our prevalence, though high, mirrors patterns seen in referral-based samples and not general populations.

Our technical error rates also resemble data from other CBCT studies. A 2025 Iranian CBCT analysis found technical errors in 44.1% of root canals: 20.6% underfilled, 8.5% missed canals, and 7.0% overfilled [84]. Similarly, a 2023 study reported underfilling, missed canals, and overfilling as the most frequent procedural errors [85]. These parallels underscore the reproducibility of core CBCT diagnostic patterns across geographies, despite differences in referral context and instrumentation techniques. [86]

Beyond the Brazilian, Kuwaiti, and Iranian reports already discussed, similar rates have been observed in other regions [86].

In European CBCT-based studies, apical periodontitis has frequently been associated with inadequate root canal filling and missed canals, supporting similar diagnostic patterns across regions [87].

A Chinese CBCT study found a significant association between missed canals and the presence of apical periodontitis in treated teeth, emphasizing the crucial role of complete canal location and obturation [88].

In a Turkish CBCT-based study, apical periodontitis was detected in 41% of endodontically treated teeth, with a significantly higher prevalence in teeth with inadequate treatment (70.8%) compared to those adequately treated (29.3%) [89]. This supports the consistent pattern reported in different populations, where underfilling and missed canals are primary risk factors for AP. Globally, meta-analyses estimate AP prevalence in root-filled teeth at approximately 39% (95% CI: 36–43%), highlighting the consistency of our findings with international trends [82].

**CBCT indications and clinical decision-making**

The use of CBCT in endodontics should follow evidence-based guidelines. According to the European Society of Endodontology, CBCT is not recommended as a routine imaging method for all patients but should be reserved for complex cases where conventional radiography is insufficient—such as persistent symptoms, suspected missed canals, or unusual anatomy [5]. The findings of this study support the diagnostic utility of CBCT in retreatment planning and underline the importance of using it selectively, balancing diagnostic benefit with radiation exposure.

The strengths of this study include the use of high-resolution CBCT imaging, which enabled a detailed and accurate assessment of both the periapical status and the quality of endodontic treatments. The application of a standardized scoring system (CBCT-PAI), combined with calibrated, independent evaluations and high inter-observer agreement, ensured methodological consistency. Additionally, the study explored multiple clinical and technical variables, providing a comprehensive understanding of factors associated with apical periodontitis.

However, some limitations should be acknowledged. The retrospective design does not allow for causal inference, and the sample—composed of patients referred for CBCT due to clinical suspicion—may not reflect the general population. Moreover, clinical symptom data were incomplete in some cases, and no histopathological confirmation was available. Despite these limitations, CBCT demonstrated a reliable and accurate method for detecting periapical lesions and evaluating obturation quality. Its ability to measure periapical dimensions and identify technical deficiencies enhances its value in complex diagnostic scenarios.

Regarding the external validity and generalizability of our findings, several factors must be considered. The sample consisted exclusively of patients referred for CBCT imaging in a specialized dental clinic, often due to suspected endodontic failure or unclear periapical status on conventional radiographs. This selective context may have introduced a referral bias, and as such, the prevalence and severity of lesions observed may not accurately reflect the general population undergoing routine dental care.

Nevertheless, the diagnostic approach and analytical methodology used in this study can be reliably extended to similar clinical contexts, especially in tertiary care or endodontic referral practices where CBCT is routinely used.

These considerations emphasize the importance of contextualizing the results and support the need for further prospective studies in more diverse populations.

**Methodological considerations and study limitations**

In this study, several methodological decisions directly contributed to the robustness of the findings. The use of high-resolution CBCT imaging, with a voxel size of 75 μm and a limited field of view (5 × 5 cm), allowed for enhanced visualization of fine periapical and root canal structures, minimizing diagnostic uncertainty—particularly in differentiating chronic and acute apical periodontitis [20,25].

This small field of view also ensured reduced radiation exposure by limiting imaging to the region of interest, while improving spatial resolution—a key advantage in endodontic diagnostics. The combination of clinical and radiographic evaluation can significantly enhance diagnostic precision [66].

CS 3D Imaging software enabled precise multi-planar assessment and standardization across observers. Although smoking was not statistically associated with periapical pathology in men, age-related trends in women suggest behavioral and psychosomatic factors may still play a role [76,78]. The exclusion of third molars, ongoing treatments, and low-quality scans likely improved internal validity, though these criteria limit generalizability [20,22].

Although the prevalence of apical lesions in our sample (46.69%) aligns with previous CBCT-based studies [40,41,42,43,44,45], these results must be interpreted within the study’s limited context. The sample does not reflect asymptomatic populations or routine dental care. Additionally, CBCT has intrinsic limitations—including increased radiation dose and image artifacts that may mimic lesions—potentially leading to false positives. These aspects should be carefully weighed when generalizing the findings.

Several methodological limitations must be acknowledged. The retrospective design and the indication-based referral to CBCT likely introduced selection bias, potentially overrepresenting symptomatic or complex cases. Consequently, the reported prevalence of apical periodontitis should be interpreted as specific to this referred population rather than as a population-based rate. The combination of clinical and radiographic evaluation has been shown to significantly enhance diagnostic precision [66].

No CBCT-PAI scores of 1–2 were observed; this truncation of the scale may bias the distribution of lesion severity and limit comparisons between small and larger lesions. The absence of low scores is likely a sampling artifact reflecting the clinical indications for CBCT referral, which predominantly targeted suspected endodontic complications or advanced periapical disease.

The observed association suggesting that chronic disease may have a greater impact was derived from tooth-level counts per patient rather than patient-centered outcomes or longitudinal lesion data, and no adjustment for potential confounders (e.g., age, comorbidities) was performed. These factors limit the strength of this inference

Inter- and intra-observer reliability was estimated on a relatively small calibration set of 20 images; although ICC values were high, the limited sample size may reduce the stability of these estimates.

An apparent paradox was observed teeth with radiographically satisfactory root fillings showed a high prevalence of apical periodontitis, whereas unsatisfactory fillings were more frequently associated with medium-to-large lesions. This may be reflected in healing apical lesions or chronic, long-standing lesions persisting despite technically adequate treatment—possibly influenced by complex anatomy, persistent extraradicular infection, or older treatments—whereas unsatisfactory fillings may more often represent acute or progressive disease. However, due to the retrospective design, detailed data on time since treatment, operator factors, and tooth-type stratification were unavailable; therefore, these associations should be interpreted with caution.

Given that CBCT examinations in this study were performed selectively in cases with complex diagnostic or treatment needs, the sample is enriched for suspected endodontic complications. This referral-based selection likely results in a higher observed prevalence of apical periodontitis compared to the general population. Therefore, the reported prevalence figures should not be interpreted as population-based rates but rather as specific to this high-risk, referred cohort.

Although a correct apical extension was defined as 0–0.5 mm from the radiographic apex, variability in the anatomical position of the apical constriction and possible measurement error due to CBCT spatial resolution could influence classification accuracy. These factors may have resulted in minor misclassification of root filling adequacy in some cases.

Analyses were performed at the tooth level without clustering adjustment; thus, statistical significance may be slightly inflated due to intra-patient correlation.

**Clinical implications and future directions**

This study confirms the strong association between apical periodontitis and inadequate root canal filling, emphasizing the importance of strict adherence to endodontic protocols. Although the analysis was limited to radiographic findings, the results support the diagnostic utility of CBCT in complex clinical scenarios—particularly retreatment cases—where conventional imaging proves inconclusive. In line with the 2023 European Society of Endodontology guidelines [5], CBCT should be employed selectively, balancing diagnostic benefit with radiation exposure.

Future prospective studies should aim to integrate CBCT assessments with clinical symptoms (e.g., pain, tenderness) and long-term tooth survival data to enhance prognostic accuracy. Additionally, larger multicenter studies are needed to validate the generalizability of these findings beyond tertiary care settings, particularly in general dental practice.

The integration of CBCT into the diagnostic workflow—when used judiciously—can improve endodontic decision-making, reduce failure rates, and support long-term tooth preservation.

## 5. Conclusions

Within the limits of this retrospective, referral-based study, CBCT proved useful in detecting apical periodontitis in root canal-treated teeth in a population enriched for complex or suspected failure cases. In this sample, 46.69% of treated teeth presented with apical periodontitis. These results should not be extrapolated to the general population but highlight the diagnostic value of CBCT when used selectively and in accordance with established guidelines. The findings also emphasize the importance of interpreting prevalence data in light of patient selection criteria and clinical indications for imaging.

## Figures and Tables

**Table 1:** Distribution of patients by gender.

Parameter	Category	Gender			*p*
		Females	Males	Total	
		145 Patients	113 Patients	258 Patients	
Age group	≤40 years old	72 (53.33%)	63 (46.67%)	135 (100%)	
		49.66%	55.75%		0.198 *
	>40 years old	73 (59.35%)	50 (40.65%)	123 (100%)	ω = 0.061
		50.34%	44.25%		
Age	Median value	44.00	37.00	-	0.129 ** r = 0.094
Medical condition	Healthy	96 (53.04%)	85 (46.96%)	181 (100%)	
		66.21%	75.22%		0.116 *
	Chronic diseases	49 (63.64%)	28 (36.36%)	77 (100%)	ω = 0.106
		33.79%	24.78%		
Harmful habits	Yes	73 (54.07%)	62 (45.93%)	135 (100%)	
		50.34%	54.87%		0.471 *
	No	72 (58.54%)	51 (41.46%)	123 (100%)	ω = 0.045
		49.66%	45.13%		
Total number of teeth	Mean ± SD	24.228 ± 6.28	25.619 ± 5.785	-	0.052 ** r = 0.121
	Median values	26.00	27.00		
Number of teeth with endodontic treatments	Mean ± SD	3.683 ± 2.92	3.027 ± 2.388	-	0.116 ** r = 0.098
	Median values	3.00	2.00		
Number of teeth with endodontic treatments and no AP	Mean ± SD	2.09 ± 2.563	1.451 ± 2.04	-	0.082 ** r = 0.108
	Median values	1.00	1.00		
Number of teeth with AP	Mean ± SD	1.6 ± 0.74	1.566 ± 0.68	-	0.874 ** r = 0.010
	Median values	1.00	1.00		
Number of teeth with acute AP	Mean ± SD	1.08 ± 0.39	1.00 ± 0.00	-	0.414 ** r = 0.051
	Median values	1.00	1.00		
Number of teeth with chronic AP	Mean ± SD	2.08 ± 0.758	2.01 ± 0.663	-	0.508 ** r = 0.041
	Median values	2.00	2.00		
Abutment teeth	Yes	56 (61.54%)	35 (38.46%)	91 (100%)	
		38.62%	30.97%		0.202 *
	No	89 (53.29%)	78 (46.71%)	167 (100%)	ω = 0.079
		61.38%	69.03%		
Intraradicular posts	Yes	55 (61.11%)	35 (38.89%)	90 (100%)	
		37.93%	30.97%		0.245 *
	No	90 (53.57%)	78 (46.43%)	168 (100%)	ω = 0.072
		62.07%	69.03%		

* Chi-Square test. ** Mann-Whitney U test. The values in grey are summed by columns.

**Table 2:** Distribution of patients by age groups.

Parameter	Category	Age Groups			*p*
		≤40 Years Old	>40 Years Old	Total	
		135 Patients	123 Patients	258 Patients	
Medical condition	Healthy	130 (71.82%)	51 (28.18%)	181 (100%)	
		96.30%	41.46%		<0.0005 *^,#^
	Chronic diseases	5 (6.49%)	72 (93.51%)	77 (100%)	ω = 0.599
		3.70%	58.54%		
Harmful habits	Yes	64 (47.41%)	71 (52.59%)	135 (100%)	
		47.41%	57.72%		0.098 *
	No	71 (57.72%)	52 (42.28%)	123 (100%)	ω = 0.103
		52.59%	42.28%		
Total number of teeth	Mean ± SD	28.98 ± 2.34	20.29 ± 5.69	-	<0.0005 **^,#^ *r* = 0.768
	Median values	29.00	21.00		
Number of teeth with endodontic treatments	Mean ± SD	2.66 ± 1.97	4.20 ± 3.17	-	<0.0005 **^,#^ *r* = 0.734
	Median values	2.00	3.00		
Number of teeth with endodontic treatments and no AP	Mean ± SD	1.20 ± 1.61	2.48 ± 2.84	-	0.001 **^,#^ *r* = 0.215
	Median values	1.00	2.00		
Number of teeth with AP	Mean ± SD	1.46 ± 0.67	1.72 ± 0.74	-	0.002 **^,#^ *r* = 0.192
	Median values	1.00	2.00		
Number of teeth with acute AP	Mean ± SD	1.00 ± 0.00	1.13 ± 0.49	-	0.036 **^,#^ *r* = 0.130
	Median values	1.00	1.00		
Number of teeth with chronic AP	Mean ± SD	1.96 ± 0.69	2.11 ± 0.73	-	0.005 **^,#^ *r* = 0.175
	Median values	2.00	2.00		
Abutment teeth	Yes	17 (18.68%)	74 (81.32%)	91 (100%)	
		12.59%	60.16%		<0.0005 *^,#^
	No	118 (70.66%)	49 (29.34%)	167 (100%)	ω = 0.497
		87.41%	39.84%		
Intraradicular posts	Yes	36 (40.00%)	54 (60.00%)	90 (100%)	
		26.67%	43.90%		0.004 *^,#^
	No	99 (58.93%)	69 (41.07%)	168 (100%)	ω = 0.181
		73.33%	56.10%		

* Chi-square test. ** Mann–Whitney U test. The values in grey are summed by columns. ^#^ Statistically significant.

**Table 3:** Distribution of patients by periapical diagnosis.

Parameter	Category	Apical Periodontitis			*p*
		Acute	Chronic	Total	
		75 Patients	183 Patients	258 Patients	
Gender	Females	45 (31.03%)	100 (68.97%)	145 (100%)	
		60.00%	54.64%		0.431 *
	Males	30 (26.55%)	83 (73.45%)	113 (100%)	ω = 0.049
		40.00%	45.36%		
Age group	≤40 years old	49 (36.30%)	86 (63.70%)	135 (100%)	
		65.33%	46.99%		0.007 *^,#^
	>40 years old	26 (21.14%)	97 (78.86%)	123 (100%)	ω = 0.167
		34.67%	53.01%		
Medical condition	Healthy	60 (33.15%)	121 (66.85%)	181 (100%)	
		80.00%	66.12%		0.027 *^,#^
	Chronic diseases	15 (19.48%)	62 (80.52%)	77 (100%)	ω = 0.138
		20.00%	33.88%		
Harmful habits	Yes	35 (25.93%)	100 (74.07%)	135 (100%)	
		46.67%	54.64%		0.244 *
	No	40 (32.52%)	83 (67.48%)	123 (100%)	ω = 0.073
		53.33%	45.36%		
Total number of teeth	Mean ± SD	26.13 ± 6.62	24.31 ± 5.805	-	0.001 **^,#^ *r* = 0.200
	Median values	28.00	26.00		
Number of teeth with endodontic treatments	Mean ± SD	1.55 ± 1.154	4.15 ± 2.807	-	<0.0005 **^,#^ *r* = 0.571
	Median values	1.00	3.00		
Number of teeth with endodontic treatments and no AP	Mean ± SD	0.53 ± 1.143	2.33 ± 2.534	-	<0.0005 **^,#^ *r* = 0.424
	Median values	0.00	2.00		
Number of teeth with AP	Mean ± SD	1.03 ± 0.231	1.81 ± 0.717	-	<0.0005 **^,#^ *r* = 0.539
	Median values	1.00	2.00		
Abutment teeth	Yes	12 (13.19%)	79 (86.81%)	91 (100%)	
		16.00%	43.17%		<0.0005 *^,#^
	No	63 (37.72%)	104 (62.28%)	167 (100%)	ω = 0.258
		84.00%	56.83%		
Intraradicular post	Yes	10 (11.11%)	80 (88.89%)	90 (100%)	
		13.33%	43.72%		<0.0005 *^,#^
	No	65 (38.69%)	103 (61.31%)	168 (100%)	ω = 0.289
		86.67%	56.28%		
AP lesion size	Small lesions	46 (26.59%)	127 (73.41%)	173 (100%)	
		61.33%	69.40%		0.211 *
	Medium-Large lesions	29 (34.12%)	56 (65.88%)	85 (100%)	ω = 0.084
		38.67%	30.60%		
Affected hemiarch	Bimaxillary	3 (3.75%)	77 (96.25%)	80 (100%)	
		4.00%	42.08%		
	Superior	39 (32.50%)	81 (67.50%)	120 (100%)	<0.0005 *^,#^
		52.00%	44.26%		ω = 0.428
	Inferior	33 (56.90%)	25 (43.10%)	58 (100%)	
		44.00%	13.66%		
Underfillings	Yes	61 (26.18%)	172 (73.82%)	233 (100%)	
		81.33%	93.99%		0.002 *^,#^
	No	14 (56.00%)	11 (44.00%)	25 (100%)	ω = 0.194
		18.67%	6.01%		
Correct obturations	Yes	9 (23.68%)	29 (76.32%)	38 (100%)	
		12.00%	15.85%		0.429 *
	No	66 (30.00%)	154 (70.00%)	220 (100%)	ω = 0.049
		88.00%	84.15%		
Overfillings	Yes	7 (12.73%)	48 (87.27%)	55 (100%)	
		9.33%	26.23%		0.003 *^,#^
	No	68 (33.5%)	135 (66.5%)	203 (100%)	ω = 0.187
		90.67%	73.77%		
Gaps	Yes	55 (29.57%)	131 (70.43%)	186 (100%)	
		73.33%	71.58%		0.776 *
	No	20 (27.78%)	52 (72.22%)	72 (100%)	ω = 0.018
		26.67%	28.42%		

* Chi-square test. ** Mann–Whitney U test. The values in grey are summed by columns. ^#^ Statistically significant.

**Table 4:** Distribution of endodontically treated teeth by the presence of AP.

Parameter	Category	Endodontically Treated Teeth			*p*
		With AP	No AP	Total	
		409 Teeth	467 Teeth	876 Teeth	
Gender	Females	232 (43.36%)	303 (56.64%)	535 (100%)	
		56.72%	64.88%		0.013 *^,#^
	Males	177 (51.91%)	164 (48.09%)	341 (100%)	ω = 0.083
		43.28%	35.12%		
Age group	≤40 years old	197 (54.72%)	163 (45.28%)	360 (100%)	
		48.17%	34.90%		<0.0005 *^,#^
	>40 years old	212 (41.09%)	304 (58.91%)	516 (100%)	ω = 0.134
		51.83%	65.10%		
Medical condition	Healthy	273 (52.10%)	251 (47.90%)	524 (100%)	
		66.75%	53.75%		<0.0005 *^,#^
	Chronic diseases	136 (38.64%)	216 (61.36%)	352 (100%)	ω = 0.132
		33.25%	46.25%		
Harmful habits	Yes	240 (46.88%)	272 (53.13%)	512 (100%)	
		58.68%	58.24%		0.896 *
	No	169 (46.43%)	195 (53.57%)	364 (100%)	ω = 0.004
		41.32%	41.76%		
Teeth type	Incisors	49 (100%)	0 (0%)	49 (100%)	
		11.98%	0%		
	Premolars	123 (43.93%)	157 (56.07%)	280 (100%)	<0.0005 *^,#^
		30.07%	33.62%		ω = 0.464
	Molars	237 (43.33%)	310 (56.67%)	547 (100%)	
		57.95%	66.38%		
Root canal filling	Underfillings	247 (56.26%)	192 (43.74%)	439 (100%)	
		60.39%	41.11%		<0.0005 *^,#^
	Correct obturations	32 (68.09%)	15 (31.91%)	47 (100%)	ω = 0.245
		7.82%	3.21%		
	Overfillings	130 (33.33%)	260 (66.67%)	390 (100%)	
		31.78%	55.67%		
Root filling quality	Satisfactory	342 (51.98%)	316 (48.02%)	658 (100%)	
		83.62%	67.67%		<0.0005 *^,#^
	Unsatisfactory	67 (30.73%)	151 (69.27%)	218 (100%)	ω = 0.184
		16.38%	32.33%		
Undetected canals	Yes	20 (95.24%)	1 (4.76%)	21 (100%)	
		4.89%	0.21%		<0.0005 *^,#^
	No	389 (45.5%)	466 (54.5%)	855 (100%)	ω = 0.153
		95.11%	99.79%		
Gaps	Yes	307 (45.55%)	367 (54.45%)	674 (100%)	
		75.06%	78.59%		0.216 *
	No	102 (50.5%)	100 (49.5%)	202 (100%)	ω = 0.052
		24.94%	21.41%		

* Chi-square test. The values in grey are summed by columns. ^#^ Statistically significant.

**Table 5:** Distribution of endodontically treated teeth by the quality of the root treatment canal.

Parameter	Category	Root Filling Quality			*p*
		Unsatisfactory	Satisfactory	Total	
		567 Teeth	309 Teeth	876 Teeth	
Gender	Females	408 (76.26%)	127 (23.74%)	535 (100%)	
		62.01%	58.26%		0.325 *
	Males	250 (73.31%)	91 (26.69%)	341 (100%)	ω = 0.033
		37.99%	41.74%		
Age group	≤40 years old	276 (76.67%)	84 (23.33%)	360 (100%)	
		41.95%	38.53%		0.375 *^,#^
	>40 years old	382 (74.03%)	134 (25.97%)	516 (100%)	ω = 0.030
		58.05%	61.47%		
Medical condition	Healthy	386 (73.66%)	138 (26.34%)	524 (100%)	
		58.66%	63.3%		0.226 *
	Chronic diseases	272 (77.27%)	80 (22.73%)	352 (100%)	ω = 0.041
		41.34%	36.7%		
Harmful habits	Yes	410 (80.08%)	102 (19.92%)	512 (100%)	
		62.31%	46.79%		<0.0005 *^,#^
	No	248 (68.13%)	116 (31.87%)	364 (100%)	ω = 0.136
		37.69%	53.21%		
Teeth type	Incisors	35 (71.43%)	14 (28.57%)	49 (100%)	
		5.32%	6.42%		
	Premolars	186 (66.43%)	94 (33.57%)	280 (100%)	<0.0005 *^,#^
		28.27%	43.12%		ω = 0.190
	Molars	437 (79.89%)	110 (20.11%)	547 (100%)	
		66.41%	50.46%		
Undetected canals	Yes	20 (95.24%)	1 (4.76%)	21 (100%)	
		3.04%	0.46%		0.031 *^,#^
	No	638 (74.62%)	217 (25.38%)	855 (100%)	ω = 0.073
		96.96%	99.54%		
Gaps	Yes	509 (75.52%)	165 (24.48%)	674 (100%)	
		77.36%	75.69%		0.612 *
	No	149 (73.76%)	53 (26.24%)	202 (100%)	ω = 0.019
		22.64%	24.31%		

* Chi-square test. The values in grey are summed by columns. ^#^ Statistically significant.

**Table 6:** Distribution of CBCT-PAI scores among endodontically treated teeth.

Parameter	Category	AP Lesion Size (PAI Score)			*p*
		Small Lesions	Medium-Large Lesions	Total	
		290 Teeth	119 Teeth	409 Teeth	
Gender	Females	169 (72.84%)	63 (27.16%)	232 (100%)	
		58.28%	52.94%		0.323 *
	Males	121 (68.36%)	56 (31.64%)	177 (100%)	ω = 0.049
		41.72%	47.06%		
Age group	≤40 years old	141 (71.57%)	56 (28.43%)	197 (100%)	
		48.62%	47.06%		0.774 *
	>40 years old	149 (70.28%)	63 (29.72%)	212 (100%)	ω = 0.014
		51.38%	52.94%		
Medical condition	Healthy	193 (70.70%)	80 (29.30%)	273 (100%)	
		66.55%	67.23%		0.895 *^,#^
	Chronic diseases	97 (71.32%)	39 (28.68%)	136 (100%)	ω = 0.007
		33.45%	32.77%		
Harmful habits	Yes	185 (77.08%)	55 (22.92%)	240 (100%)	
		63.79%	46.22%		0.001 *^,#^
	No	105 (62.13%)	64 (37.87%)	169 (100%)	ω = 0.162
		36.21%	53.78%		
AP type	Acute	48 (62.34%)	29 (37.66%)	77 (100%)	
		16.55%	24.37%		0.066 *
	Chronic	242 (72.89%)	90 (27.11%)	332 (100%)	ω = 0.091
		83.45%	75.63%		
Total number of teeth	Mean ± SD	24.428 ± 5.984	24.286 ± 6.683	-	0.777 ** r = 0.014
	Median values	26.00	26.00		
Number of teeth with endodontic treatments	Mean ± SD	4.345 ± 3.096	3.529 ± 2.459	-	0.017 **^,#^ *r* = 0.118
	Median values	3.00	3.00		
Number of teeth with endodontic treatments and no AP	Mean ± SD	2.334 ± 2.783	1.891 ± 2.062	-	0.491 ** r = 0.034
	Median values	2.00	1.00		
Abutment teeth	Yes	127 (73.41%)	46 (26.59%)	173 (100%)	
		43.79%	38.66%		0.339 *
	No	163 (69.07%)	73 (30.93%)	236 (100%)	ω = 0.047
		56.21%	61.34%		
Intraradicular post	Yes	128 (74.42%)	44 (25.58%)	172 (100%)	
		44.14%	36.97%		0.183 *
	No	162 (68.35%)	75 (31.65%)	237 (100%)	ω = 0.066
		55.86%	63.03%		
Teeth type	Incisors	26 (53.06%)	23 (46.94%)	49 (100%)	
		8.97%	19.33%		
	Premolars	84 (68.29%)	39 (31.71%)	123 (100%)	0.004 *
		28.97%	32.77%		ω = 0.163
	Molars	180 (75.95%)	57 (24.05%)	237 (100%)	
		62.07%	47.9%		
Affected hemiarch	Bimaxillary	118 (73.29%)	43 (26.71%)	161 (100%)	
		40.69%	36.13%		0.373 *
	Superior	125 (71.43%)	50 (28.57%)	175 (100%)	ω = 0.069
		43.10%	42.02%		
	Inferior	47 (64.38%)	26 (35.62%)	73 (100%)	
		16.21%	21.85%		
Treatment quality	Underfillings	198 (80.16%)	49 (19.84%)	247 (100%)	
		68.28%	41.18%		<0.0005 *^,#^
	Correct	21 (65.63%)	11 (34.38%)	32 (100%)	ω = 0.259
		7.24%	9.24%		
	Overfillings	71 (54.62%)	59 (45.38%)	130 (100%)	
		24.48%	49.58%		
Quality of root canal filling	Satisfactory	257 (75.15%)	85 (24.85%)	342 (100%)	
		88.62%	71.43%		<0.0005 *^,#^
	Unsatisfactory	33 (49.25%)	34 (50.75%)	67 (100%)	ω = 0.211
		11.38%	28.57%		
Undetected canals	Yes	16 (80.00%)	4 (20.00%)	20 (100%)	
		5.52%	3.36%		0.358 *
	No	274 (70.44%)	115 (29.56%)	389 (100%)	ω = 0.045
		94.48%	96.64%		
Gaps	Yes	224 (72.96%)	83 (27.04%)	307 (100%)	
		77.24%	69.75%		0.112 *
	No	66 (64.71%)	36 (35.29%)	102 (100%)	ω = 0.074
		22.76%	30.25%		

* Chi-square test. ** Mann–Whitney U test. The values in grey are summed by columns. ^#^ Statistically significant.

**Table 7:** Summary of key associations with apical periodontitis prevalence. OR = Odds ratio; CI = Confidence interval.

Factor	Odds Ratio	95% CI		*p* *
		Lower	Upper	
Gender (Female/Male)	0.709	0.540	0.932	0.013
Older age (≤40 years old/>40 years old)	1.733	1.321	2.274	<0.0005
Medical conditions (Yes/No)	1.727	1.313	2.273	<0.0005
Poor root canal filling quality (Unsatisfactory/Satisfactory)	2.439	1.761	3.379	<0.0005
Chronic vs. acute lesions (Acute/Chronic)	2.476	1.646	3.723	<0.0005

* Chi-square test.

## Data Availability

The authors declare that the data from this research are available from the corresponding authors upon reasonable request.
